# An Essay Towards a Correct Theory of the Nervous System

**Published:** 1844-07

**Authors:** 


Art. II.—*An Essay towards a Correct Theory of the Nervous System.* By J. Harrison, m.d. &c.—*Philadelphia,* 1844. 8vo, pp. 292.

The leading idea of this work originated in a passage in one of Dr. Stokes's lectures on medicine; in which it is suggested that the morbid condition of the nervous system in many of the neuroses, in which no appreciable change can be discovered, may be analogous to those Chemical phenomena, which are comprehended under the term *isomerism.* As in chemistry many bodies having the same constitution, (so far as the *number* of their component atoms is concerned,) possess totally different properties (resulting, it would seem from differences in the *arrangement* of their atoms,) so it may be imagined that a difference in the arrangement of the molecules of the component parts of the nerves, or their centres, may produce new modifications of their properties, without making any distinct change in their nature, or adding or abstracting a single organic molecule. This idea is expanded by Dr. Harrison with considerable acuteness and sagacity. He is evidently a man of great powers of observation and of reasoning; and we are disposed to consider many of his views as much more philosophical than speculations of this kind usually are. The author's clearness of thought and expression are well displayed in his examination of the electric theory of nervous action, and in his disquisition on life, which are contained in the Appendix. The chief objection we have to bring against the treatise, is its diffuseness. A large mass of well-known physiological facts is brought together, with the slightest possible connexion, often with none at all that we can discover; so that it is not always easy to discern the novelty of conception, which Dr. Harrison regards his views as possessing. We question whether the doctrine would be ever capable of *proof*, even with means of investigation far more refined than any which we at present possess; and we are inclined to believe, that it would be better to direct our attention to the sedulous inquiry into those alterations which we *can* appreciate. Many recent observations concur in leading to the belief, that a large proportion of those convulsive disorders of the nervous system, which are at present inexplicable by any known causes, are due to a disordered condition of the blood, resulting from an insufficient separation of the products of excretion, especially *urea;* and we would earnestly exhort those of our readers, who have attained sufficient proficiency in proximate organic analysis to conduct such inquiries, to test the condition of the blood and urine in all cases of obstinate convulsive diseases, which seem referrible to a *general* disordered state of the nervous system,—those especially which are commonly designated as the severer forms of hysteria.

